# Supplementary material for: Some simulations of age-period-cohort analysis applying Bayesian regularization: Conditions for using random walk model
Source: PLoS One. 2025 Aug 8;20(8):e0329223. doi: 10.1371/journal.pone.0329223 (PMC12334005; doi:10.1371/journal.pone.0329223)
Supplement: S1 Fig — (PDF) [file pone.0329223.s001.pdf]

S1 Fig. Comparison of the three models' estimates (Simulation 1)

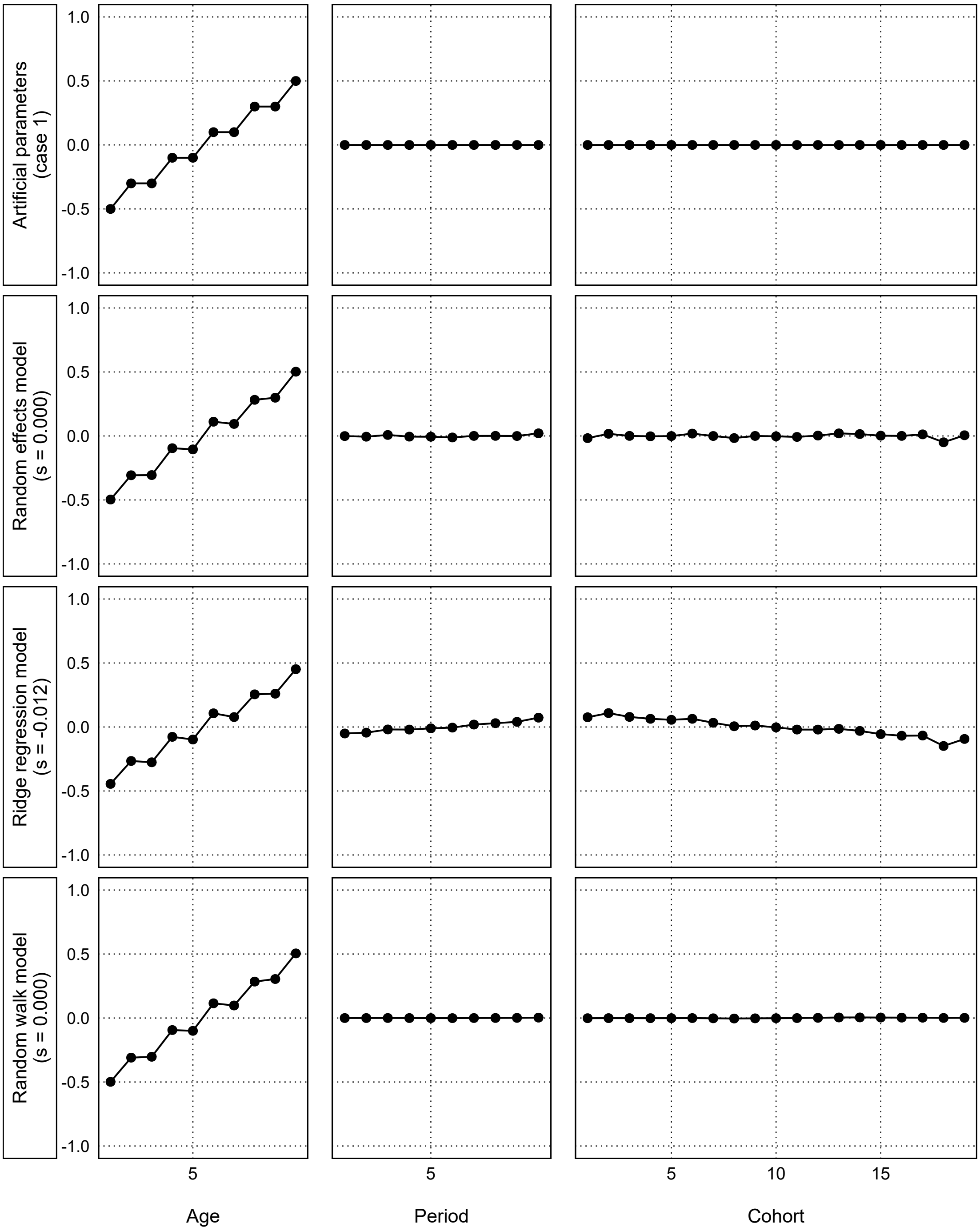

S1 Fig.1 Comparison of the three models' estimates (case 1)

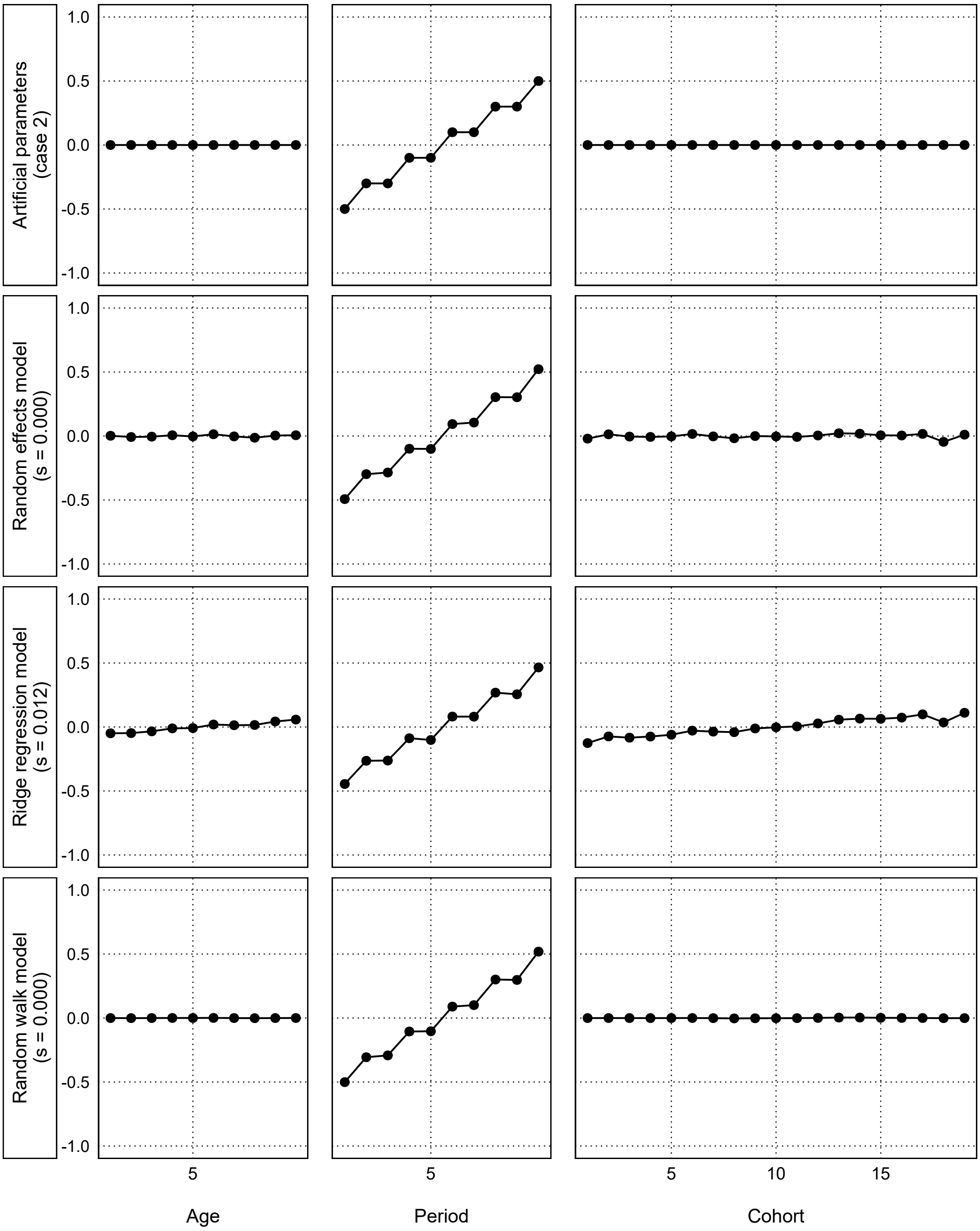

S1 Fig.2 Comparison of the three models' estimates (case 2)

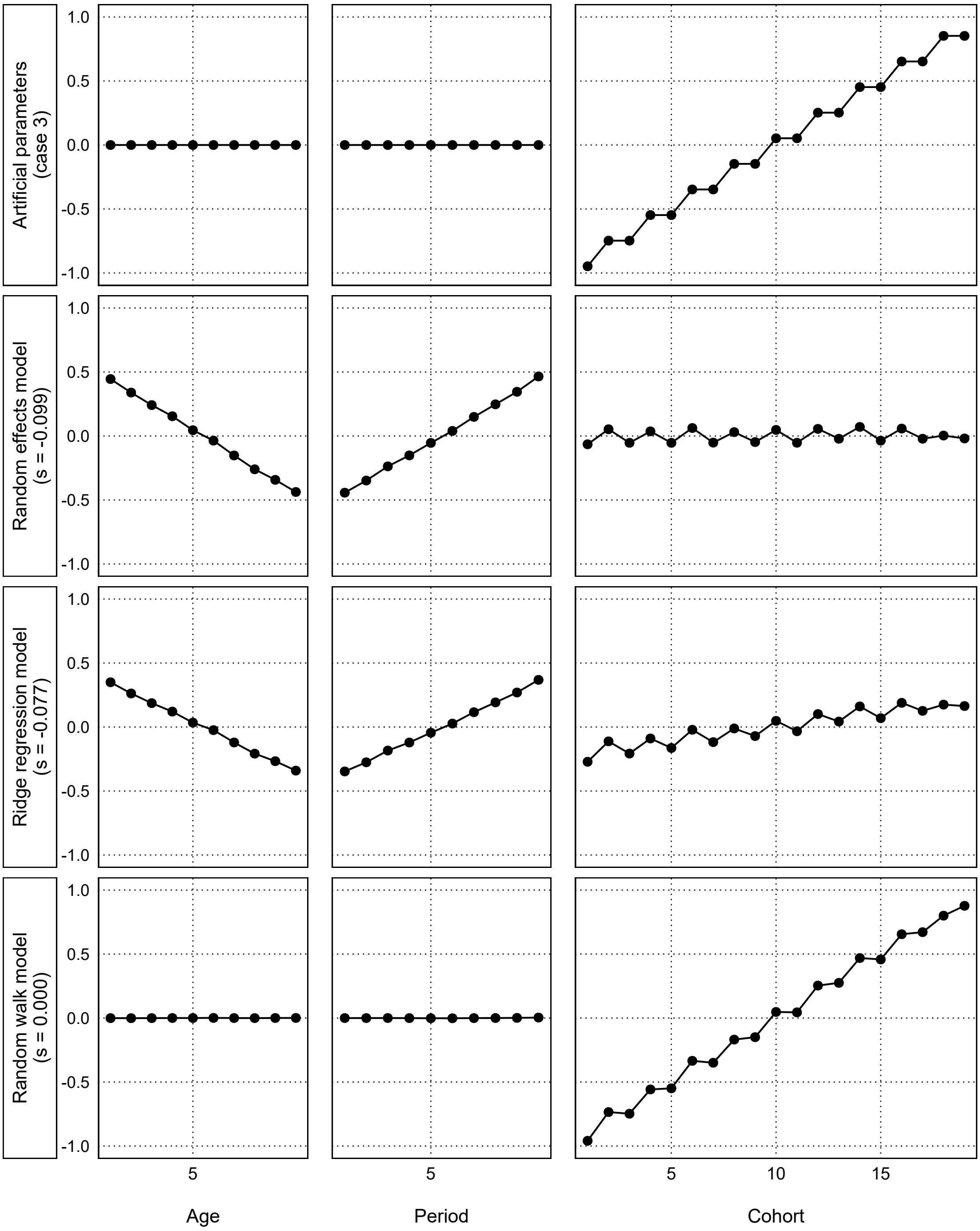

S1 Fig.3 Comparison of the three models' estimates (case 3)

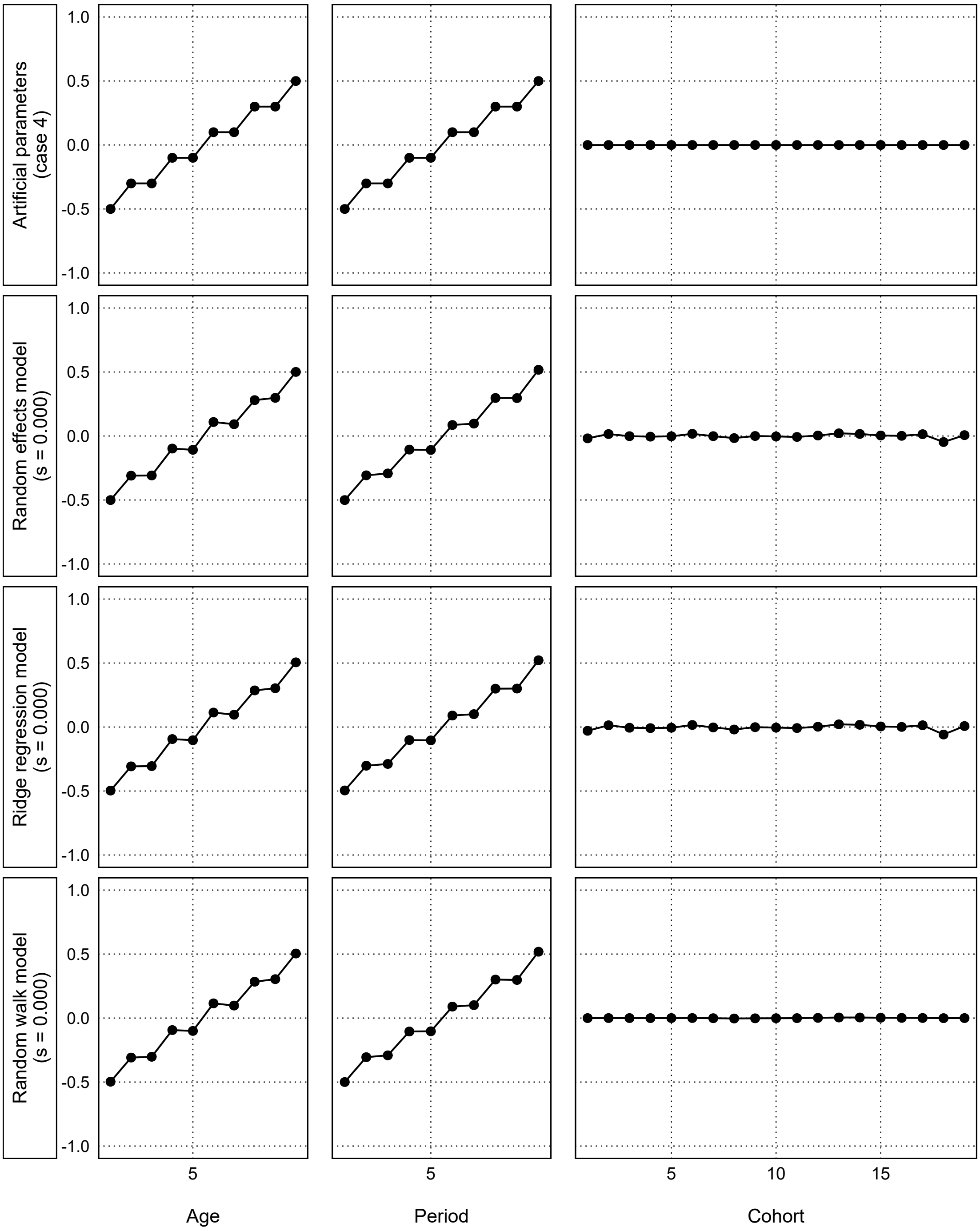

S1 Fig.4 Comparison of the three models' estimates (case 4)

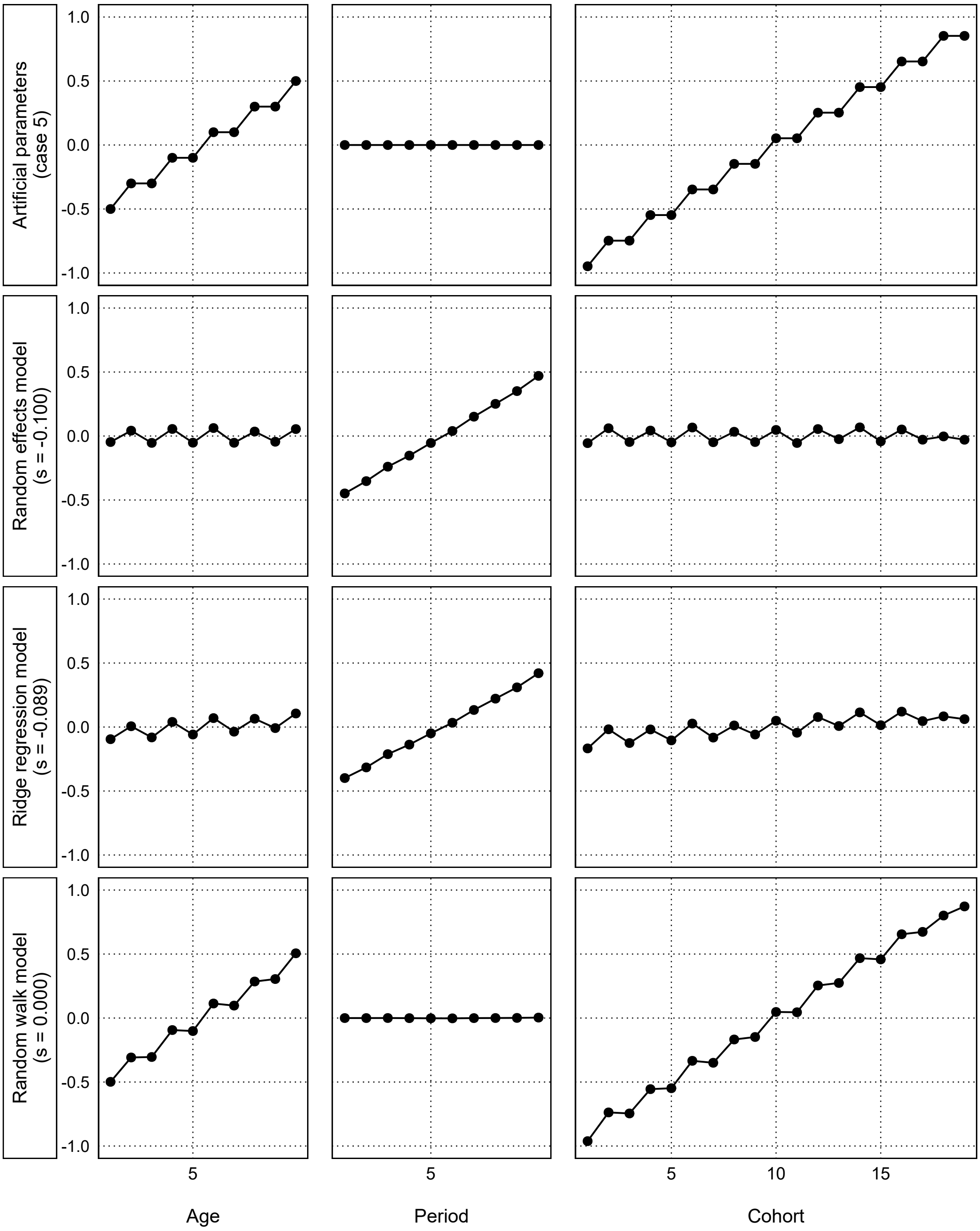

S1 Fig.5　Comparison of the three models' estimates (case 5)

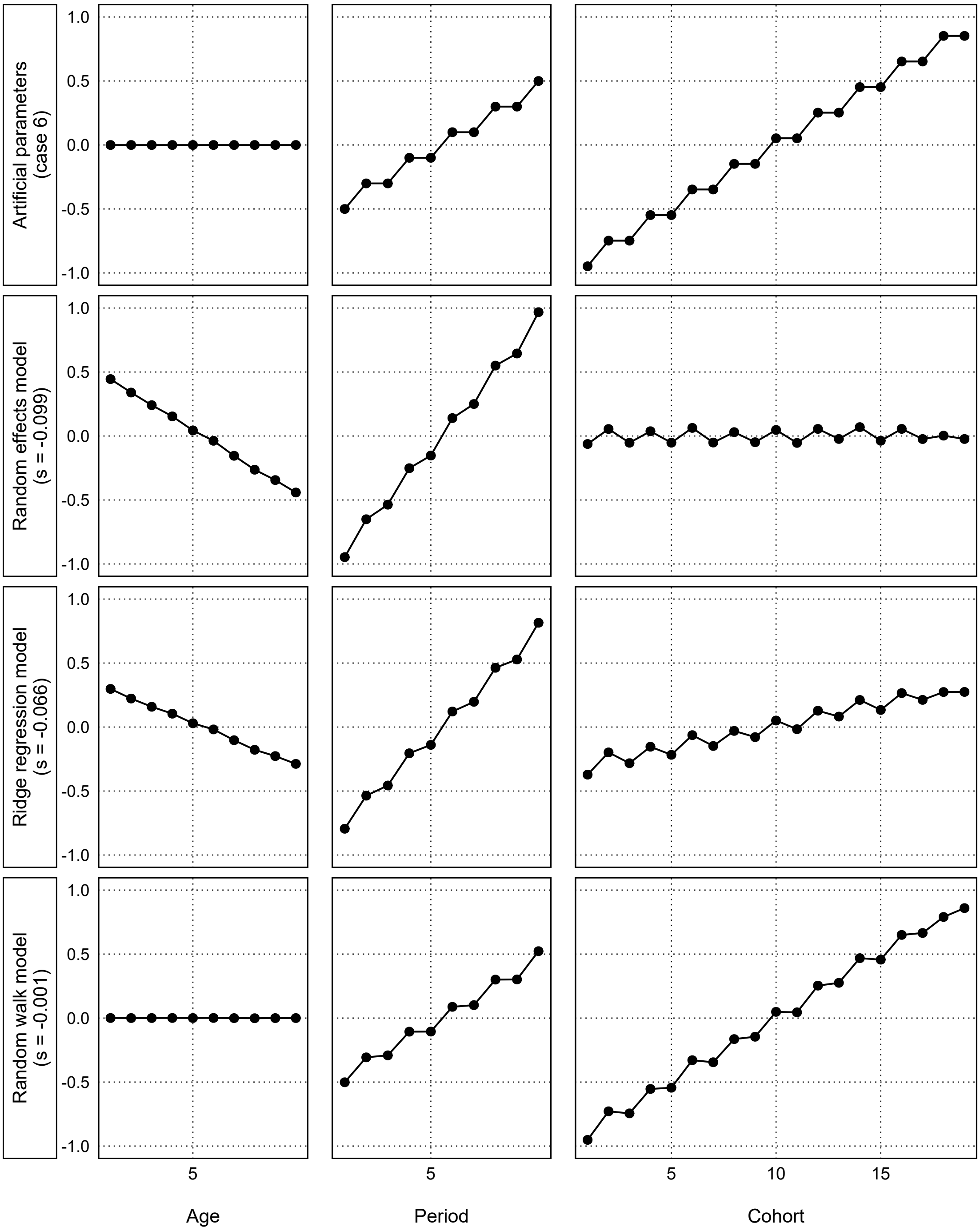

S1 Fig.6 Comparison of the three models' estimates (case 6)

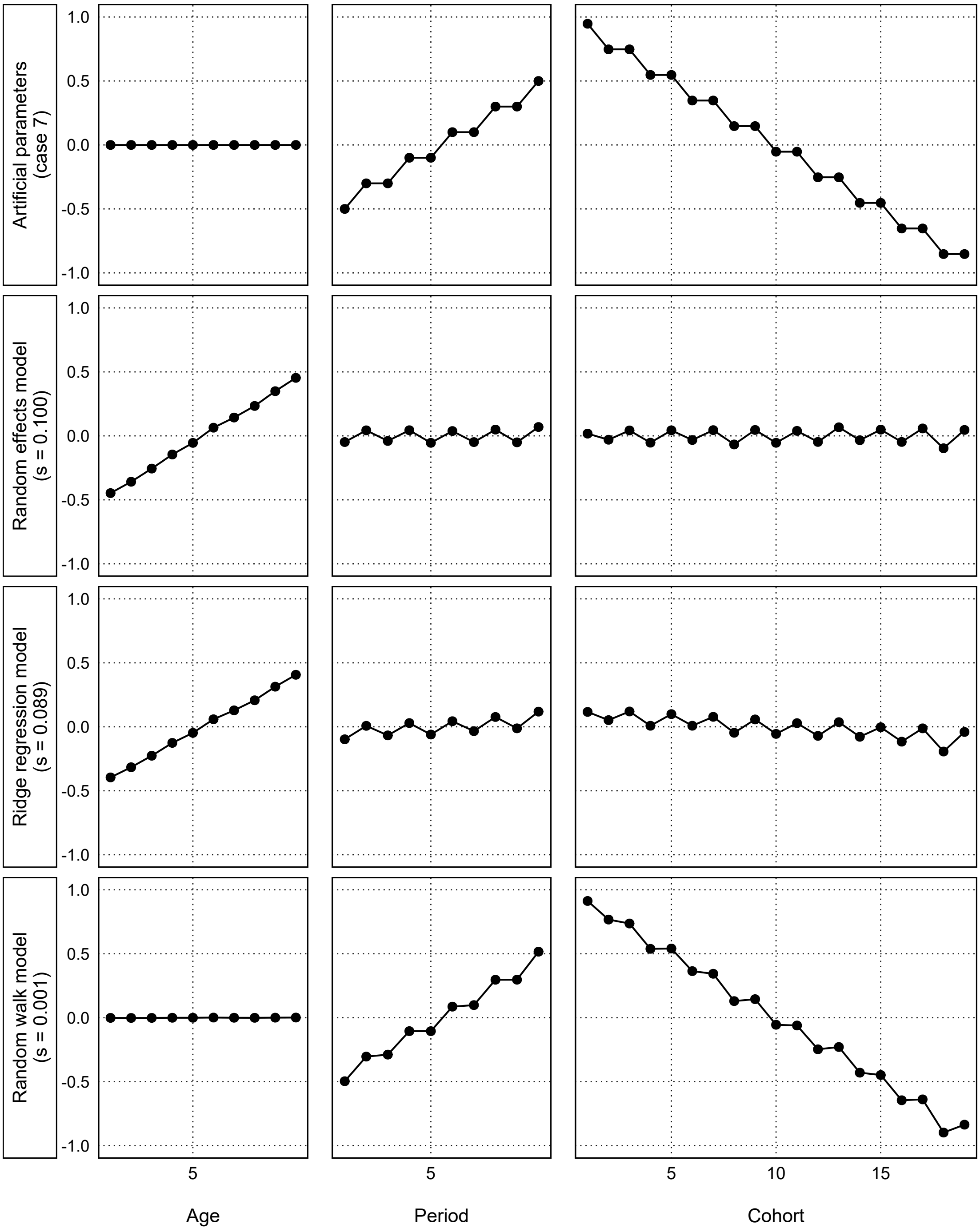

S1 Fig.7 Comparison of the three models' estimates (case 7)

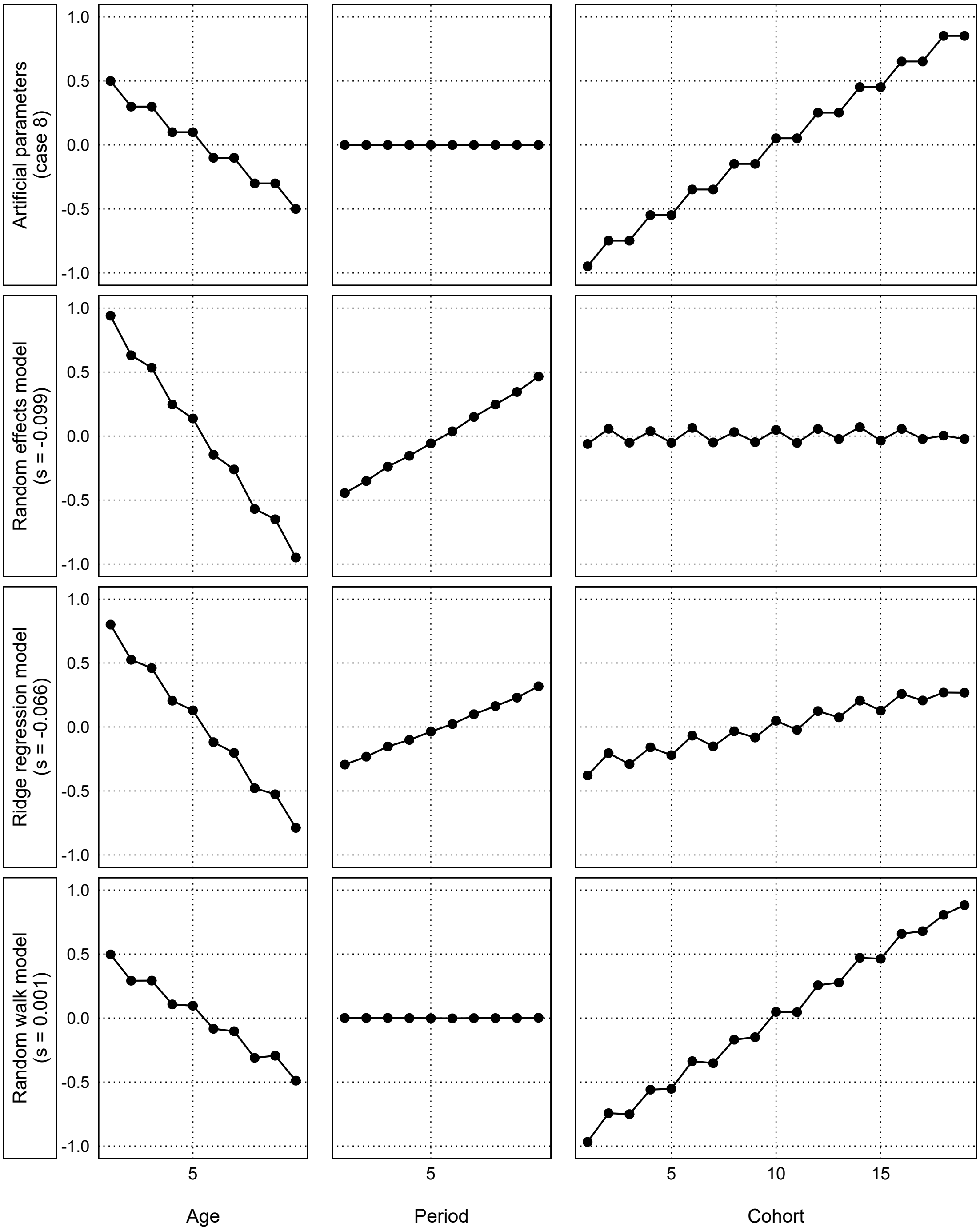

S1 Fig.8 Comparison of the three models' estimates (case 8)

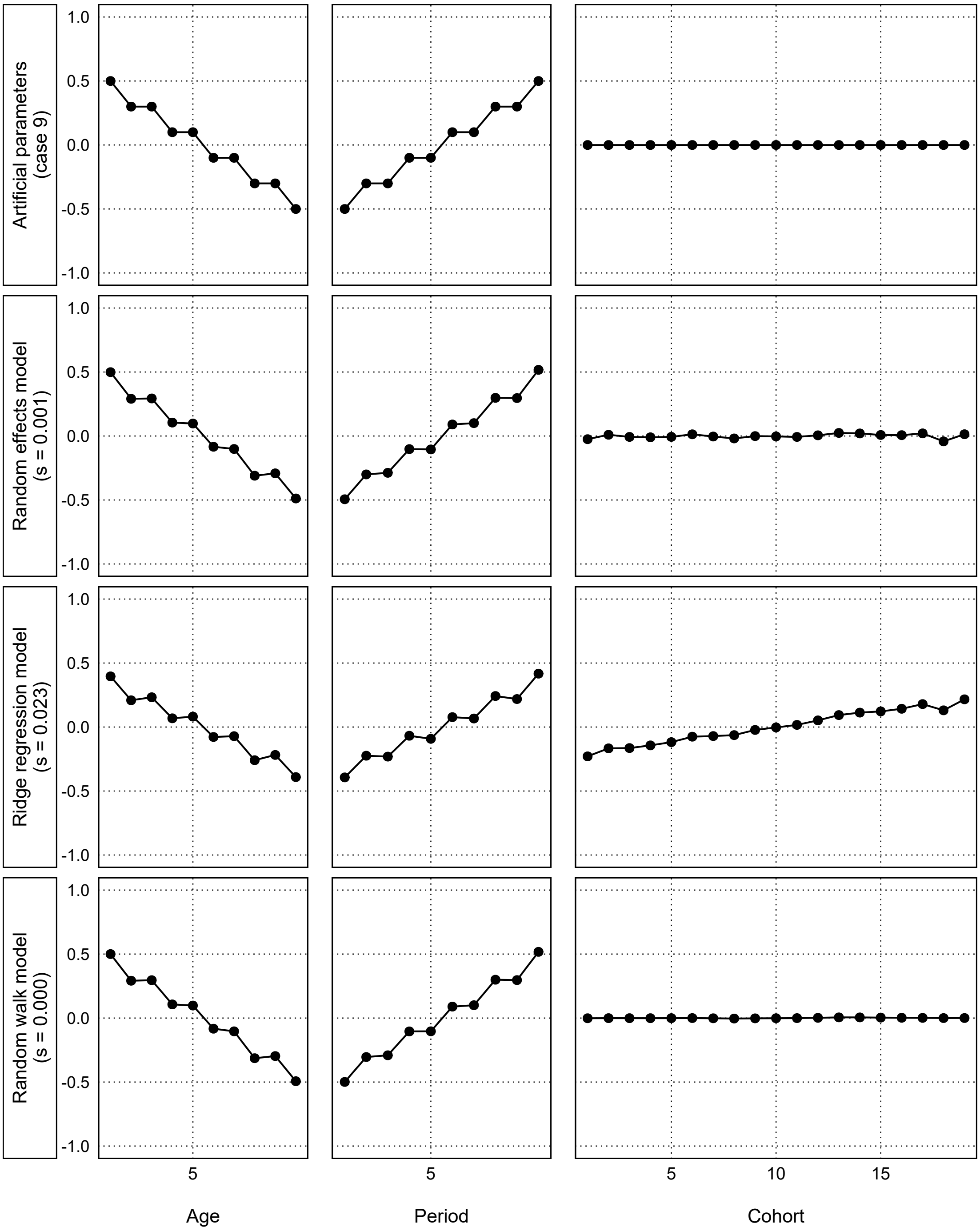

S1 Fig.9 Comparison of the three models' estimates (case 9)

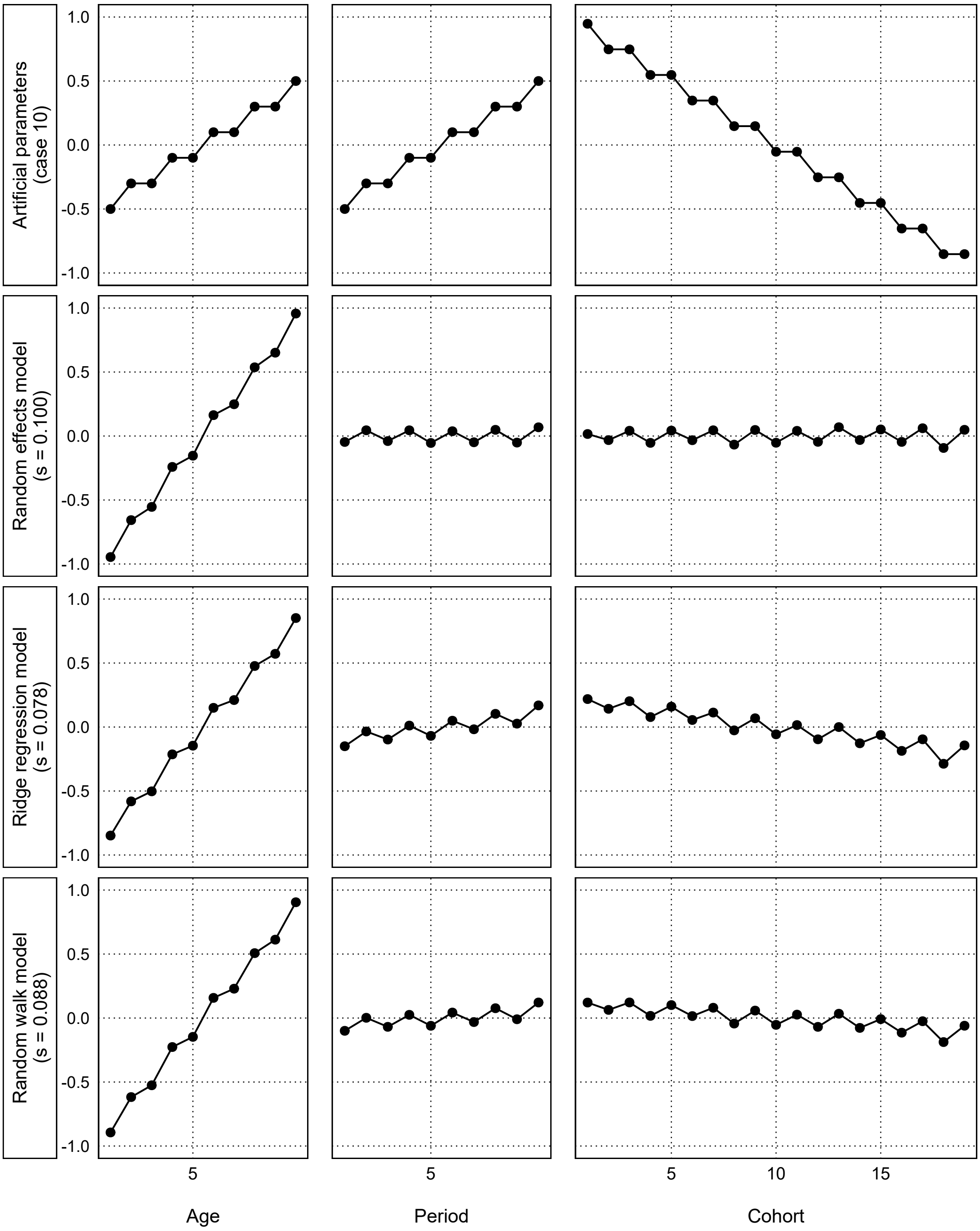

S1 Fig.10 Comparison of the three models' estimates (case 10)

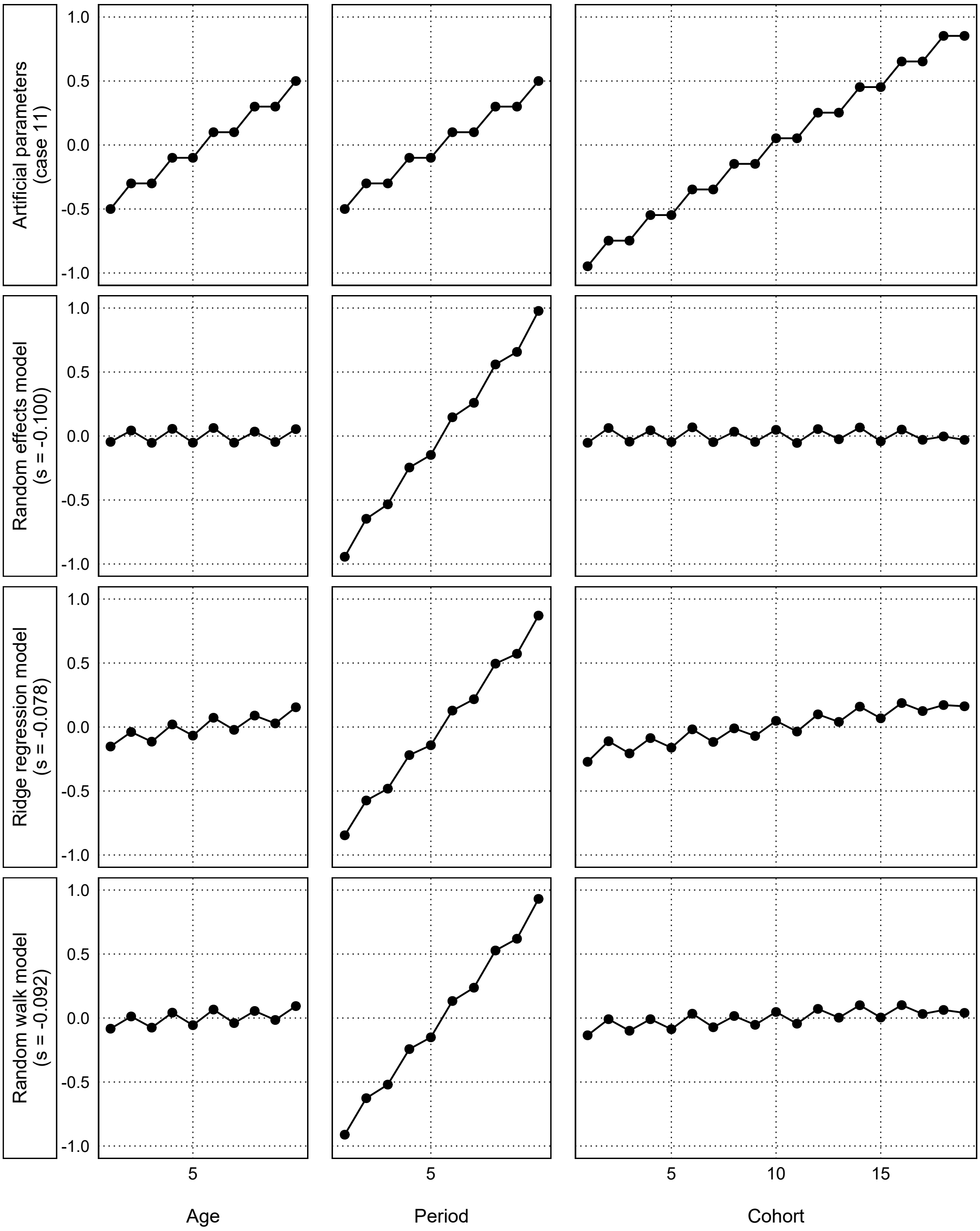

S1 Fig.11 Comparison of the three models' estimates (case 11)

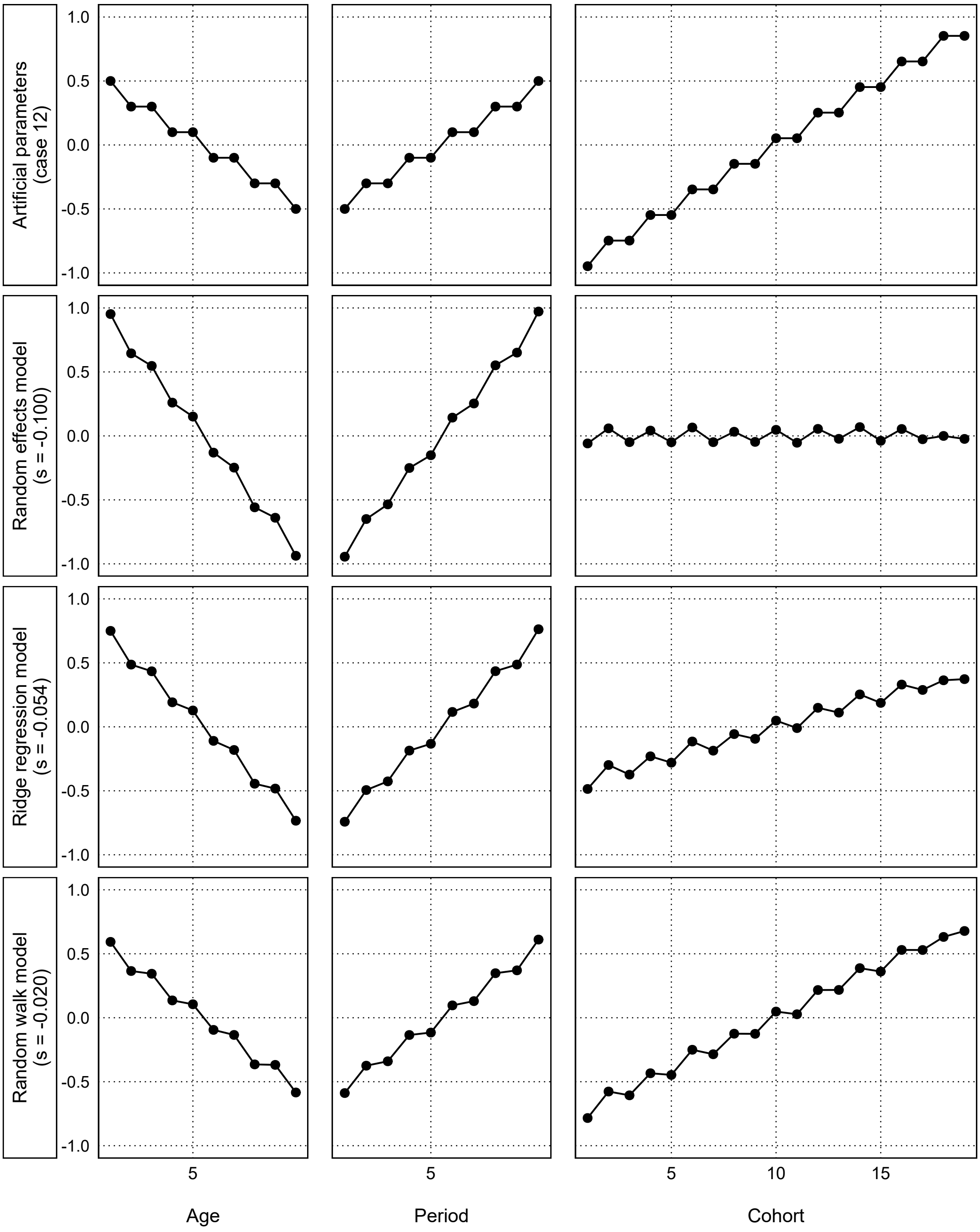

S1 Fig.12 Comparison of the three models' estimates (case 12)

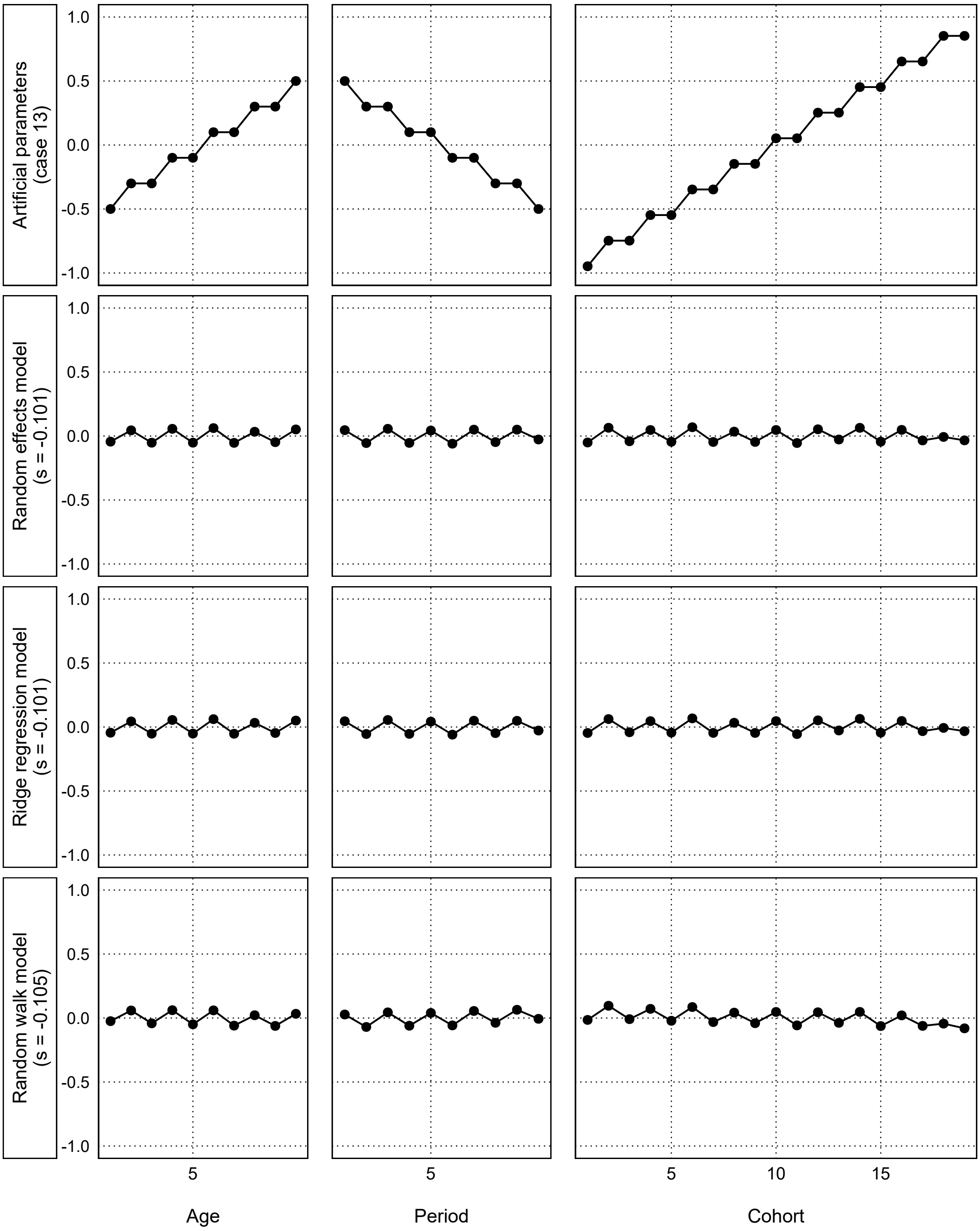

S1 Fig.13 Comparison of the three models' estimates (case 13)
