# Supplementary material for: Some simulations of age-period-cohort analysis applying Bayesian regularization: Conditions for using random walk model
Source: PLoS One. 2025 Aug 8;20(8):e0329223. doi: 10.1371/journal.pone.0329223 (PMC12334005; doi:10.1371/journal.pone.0329223)
Supplement: S1 Appendix — (PDF) [file pone.0329223.s002.pdf]

S1 Appendix. Stan codes to implement Bayesian regularization models

Random effects model (RE_Normal_APC.stan)

```
data {
  int N;
  int I;
  int J;
  int K;
  int L;
  matrix[N, L]  X;
  vector[N]     Y;
  real<lower=0> Min;
}

parameters {
  real<lower=0> raw_A;
  real<lower=0> raw_P;
  real<lower=0> raw_C;
  vector[I]     std_A;
  vector[J]     std_P;
  vector[K]     std_C;
  real<lower=0> sigma;
  real          b_0;
}

transformed parameters {
  vector[I]     b_A;
  vector[J]     b_P;
  vector[K]     b_C;
  vector[I+J]   b_AP;
  vector[L]     b;
  real<lower=0> sigma_A;
  real<lower=0> sigma_P;
  real<lower=0> sigma_C;

  sigma_A = raw_A + Min;
  sigma_P = raw_P + Min;
  sigma_C = raw_C + Min;

  b_A = sigma_A * std_A;
  b_P = sigma_P * std_P;
  b_C = sigma_C * std_C;

  b_AP = append_row(b_A,  b_P);
  b    = append_row(b_AP, b_C);
}

model {
  target += std_normal_lpdf(std_A);
  target += std_normal_lpdf(std_P);
  target += std_normal_lpdf(std_C);

  target += normal_id_glm_lpdf(Y | X, b_0, b, sigma);
}
```

Ridge regression model (`RR_Normal_APC.stan`)

```
data {
  int N;
  int I;
  int J;
  int K;
  int L;
  matrix[N, L] X;
  vector[N]    Y;
}

parameters {
  vector[I]      std_A;
  vector[J]      std_P;
  vector[K]      std_C;
  real<lower=0> sigma;
  real<lower=0> lambda;
  real           b_0;
}

transformed parameters {
  vector[I]   b_A;
  vector[J]   b_P;
  vector[K]   b_C;
  vector[I+J] b_AP;
  vector[L]   b;

  b_A = lambda * std_A;
  b_P = lambda * std_P;
  b_C = lambda * std_C;

  b_AP = append_row(b_A,  b_P);
  b    = append_row(b_AP, b_C);
}

model {
  target += std_normal_lpdf(std_A);
  target += std_normal_lpdf(std_P);
  target += std_normal_lpdf(std_C);

  target += normal_id_glm_lpdf(Y | X, b_0, b, sigma);
}
```

Random walk model (RW_Normal_APC.stan)

```
data {
  int N;
  int I;
  int J;
  int K;
  int L;
  matrix[N, L] X;
  vector[N]    Y;
}

parameters {
  vector[I-1]   std_A;
  vector[J-1]   std_P;
  vector[K-1]   std_C;
  real<lower=0> sigma;
  real<lower=0> sigma_A;
  real<lower=0> sigma_P;
  real<lower=0> sigma_C;
  real          b_0;
}

transformed parameters {
  vector[I-1] d_A;
  vector[I-1] tmp_d_A;
  vector[I]   b_A;
  vector[J-1] d_P;
  vector[J-1] tmp_d_P;
  vector[J]   b_P;
  vector[K-1] d_C;
  vector[K-1] tmp_d_C;
  vector[K]   b_C;
  vector[I+J] b_AP;
  vector[L]   b;

  // Age
  d_A = sigma_A * std_A;

  for (a in 1:(I-1)) {
    tmp_d_A[a] = (I-a) * d_A[a];
  }

  b_A[1] = -(1.0/I) * sum(tmp_d_A);

  for (i in 2:I) {
    b_A[i] = b_A[1] + sum(d_A[1:(i-1)]);
  }

  // Period
  d_P = sigma_P * std_P;

  for (p in 1:(J-1)) {
    tmp_d_P[p] = (J-p) * d_P[p];
  }

  b_P[1] = -(1.0/J) * sum(tmp_d_P);

  for (j in 2:J) {
    b_P[j] = b_P[1] + sum(d_P[1:(j-1)]);
  }
```

```
  // Cohort
  d_C = sigma_C * std_C;

  for (c in 1:(K-1)) {
    tmp_d_C[c] = (K-c) * d_C[c];
  }

  b_C[1] = -(1.0/K) * sum(tmp_d_C);

  for (k in 2:K) {
    b_C[k] = b_C[1] + sum(d_C[1:(k-1)]);
  }


  b_AP = append_row(b_A,  b_P);
  b    = append_row(b_AP, b_C);
}

model {
  target += std_normal_lpdf(std_A);
  target += std_normal_lpdf(std_P);
  target += std_normal_lpdf(std_C);

  target += normal_id_glm_lpdf(Y | X, b_0, b, sigma);
}
```
