# Supplementary material for: Some simulations of age-period-cohort analysis applying Bayesian regularization: Conditions for using random walk model
Source: PLoS One. 2025 Aug 8;20(8):e0329223. doi: 10.1371/journal.pone.0329223 (PMC12334005; doi:10.1371/journal.pone.0329223)
Supplement: S2 Appendix — (PDF) [file pone.0329223.s003.pdf]

S2 Appendix. R codes to reproduce the systematic simulation

Simulation 1 (case 8)

```
###### Set artificial parameters ######------------

library(rstan)

# case 8

I <- 10;  J <- 10;  K <- I+J-1;  L <- I+J+K
Times <- 10;  N <- I*J*Times;  gamma <- 0.1

beta_A_L <- -0.1;  beta_A_NL <- -0.05
beta_P_L <-  0.0;  beta_P_NL <-  0.00
beta_C_L <-  0.1;  beta_C_NL <-  0.05



###### Generate artificial data  ######------------

beta_A <- numeric(I);  beta_P <- numeric(J);  beta_C <- numeric(K)

for (i in 1:I) {
  beta_A[i] <- - (beta_A_NL / (2 * I)) * (cos(pi * I) -1) +
    beta_A_L * (i - (I+1)/2) + beta_A_NL * cos(pi * i)
}

for (j in 1:J) {
  beta_P[j] <- - (beta_P_NL / (2 * J)) * (cos(pi * J) -1) +
    beta_P_L * (j - (J+1)/2) + beta_P_NL * cos(pi * j)
}

for (k in 1:K) {
  beta_C[k] <- - (beta_C_NL / (2 * K)) * (cos(pi * K) -1) +
    beta_C_L * (k - (K+1)/2) + beta_C_NL * cos(pi * k)
}



Index_A <- rep(rep(1:I, times = J), times = Times)
Index_P <- rep(rep(1:J, each  = I), times = Times)
Index_C <- Index_P - Index_A + I  # k = j-i+I



X_A <- numeric();  X_P <- numeric();  X_C <- numeric()

for (i in 1:I) {
  x_A <- ifelse(Index_A == i, 1, 0);  X_A <- cbind(X_A, x_A)
}

for (j in 1:J) {
  x_P <- ifelse(Index_P == j, 1, 0);  X_P <- cbind(X_P, x_P)
}

for (k in 1:K) {
  x_C <- ifelse(Index_C == k, 1, 0);  X_C <- cbind(X_C, x_C)
}
```

```
X <- cbind(X_A, X_P, X_C)  # Design matrix
beta <- c(beta_A, beta_P, beta_C)

set.seed(1234)
Y <- as.vector(X %*% beta + rnorm(N, 0, gamma))



###### Execute Bayesian APC analysis ######------------

# Random effects model

StanData <- list(Y=Y, X=X, N=N, I=I, J=J, K=K, L=L, Min=0.05)

Pars <- c("b_0", "b_A", "b_P", "b_C",
          "sigma", "sigma_A", "sigma_P", "sigma_C")

Model_RE <- stan_model(file= "RE_Normal_APC.stan")

Result_RE <- sampling(
  Model_RE, data = StanData, pars = Pars, seed = 1234,
  chains = 4, iter = 2000, warmup = 500, thin = 3,
  control = list(adapt_delta = 0.94, max_treedepth = 12))



# Ridge regression model

StanData <- list(Y=Y, X=X, N=N, I=I, J=J, K=K, L=L)

Pars <- c("b_0", "b_A", "b_P", "b_C", "sigma", "lambda")

Model_RR <- stan_model(file= "RR_Normal_APC.stan")

Result_RR <- sampling(
  Model_RR, data = StanData, pars = Pars, seed = 1234,
  chains = 4, iter = 2000, warmup = 500, thin = 3,
  control = list(adapt_delta = 0.94, max_treedepth = 12))



# Random walk model

StanData <- list(Y=Y, X=X, N=N, I=I, J=J, K=K, L=L)

Pars <- c("b_0", "b_A", "b_P", "b_C",
          "sigma", "sigma_A", "sigma_P", "sigma_C")

Model_RW <- stan_model(file= "RW_Normal_APC.stan")

Result_RW <- sampling(
  Model_RW, data = StanData, pars = Pars, seed = 1234,
  chains = 4, iter = 2000, warmup = 500, thin = 3,
  control = list(adapt_delta = 0.94, max_treedepth = 12))
```

Simulation 3 (Random walk model)

```
###### Set ######------------

library(rstan)

I <- 10;  J <- 10;  K <- I+J-1;
L <- I+J+K;  N <- I*J*10;  gamma <- 0.1



###### Define systematic simulation  ######------------

SystematicSimulation_APC <- function(
    beta_A_L, beta_A_NL,
    beta_P_L, beta_P_NL,
    beta_C_L, beta_C_NL) {


  ### Generate artificial data

  beta_A <- numeric(I);  beta_P <- numeric(J);  beta_C <- numeric(K)

  for (i in 1:I) {
    beta_A[i] <- - (beta_A_NL / (2 * I)) * (cos(pi * I) -1) +
      beta_A_L * (i - (I+1)/2) + beta_A_NL * cos(pi * i)
  }

  for (j in 1:J) {
    beta_P[j] <- - (beta_P_NL / (2 * J)) * (cos(pi * J) -1) +
      beta_P_L * (j - (J+1)/2) + beta_P_NL * cos(pi * j)
  }

  for (k in 1:K) {
    beta_C[k] <- - (beta_C_NL / (2 * K)) * (cos(pi * K) -1) +
      beta_C_L * (k - (K+1)/2) + beta_C_NL * cos(pi * k)
  }


  Index_A <- rep(rep(1:I, times = J), times = 10)
  Index_P <- rep(rep(1:J, each  = I), times = 10)
  Index_C <- Index_P - Index_A + I # k = j-i+I


  X_A <- numeric(); X_P <- numeric(); X_C <- numeric()

  for (i in 1:I) {
    x_A <- ifelse(Index_A == i, 1, 0); X_A <- cbind(X_A, x_A)
  }

  for (j in 1:J) {
    x_P <- ifelse(Index_P == j, 1, 0); X_P <- cbind(X_P, x_P)
  }

  for (k in 1:K) {
    x_C <- ifelse(Index_C == k, 1, 0); X_C <- cbind(X_C, x_C)
  }

  X <- cbind(X_A, X_P, X_C)
  beta <- c(beta_A, beta_P, beta_C)
```

```
  set.seed(1234)
  Y <- as.vector(X %*% beta + rnorm(N, 0, gamma))



  ### Execute Bayesian APC analysis

  # Random walk model

  StanData <- list(Y=Y, X=X, N=N, I=I, J=J, K=K, L=L)

  Pars <- c("b_0", "b_A", "b_P", "b_C",
            "sigma", "sigma_A", "sigma_P", "sigma_C")

  Model_RW <- stan_model(file= "Model/RW_Normal_APC.stan")

  Result_RW <- sampling(
    Model_RW, data = StanData, pars = Pars, seed = 1234,
    chains = 4, iter = 2000, warmup = 500, thin = 3,
    control = list(adapt_delta = 0.94, max_treedepth = 12))



  ### Calculate slope

  beta <- c(beta_A, beta_P, beta_C)

  v_A <-   1:I - (I+1)/2
  v_P <- -(1:J - (J+1)/2)
  v_C <-   1:K - (K+1)/2
  v <- c(v_A, v_P, v_C)


  RW_A <- summary(Result_RW)$summary[paste0("b_A[",1:I,"]"),"50%"]
  RW_P <- summary(Result_RW)$summary[paste0("b_P[",1:J,"]"),"50%"]
  RW_C <- summary(Result_RW)$summary[paste0("b_C[",1:K,"]"),"50%"]
  b_RW <- c(RW_A, RW_P, RW_C)

  s_RW <- (v %*% (b_RW - beta)) / (v %*% v)

  Rhat <- max(
      summary(Result_RW)$summary[, "Rhat"], na.rm = TRUE
  )

  return(c(s_RW, Rhat))
}





###### Execute simulation ######------------

Times <- 500
Result <- matrix(0, nrow = Times, ncol = 2)


set.seed(1234)

beta_A_NL <- rnorm(Times, 0, 0.1)
beta_P_NL <- rnorm(Times, 0, 0.1)
beta_C_NL <- rnorm(Times, 0, 0.1)
```

```
beta_A_L <-  rnorm(Times, 0, abs(beta_A_NL))
beta_P_L <-  rnorm(Times, 0, abs(beta_P_NL))
beta_C_L <-  rnorm(Times, 0, abs(beta_C_NL))



for (n in 1:Times) {
  Result[n, ] <- SystematicSimulation_APC(
    beta_A_L = beta_A_L[n], beta_A_NL = beta_A_NL[n],
    beta_P_L = beta_P_L[n], beta_P_NL = beta_P_NL[n],
    beta_C_L = beta_C_L[n], beta_C_NL = beta_C_NL[n])
}
```
