# Supplementary material for: Some simulations of age-period-cohort analysis applying Bayesian regularization: Conditions for using random walk model
Source: PLoS One. 2025 Aug 8;20(8):e0329223. doi: 10.1371/journal.pone.0329223 (PMC12334005; doi:10.1371/journal.pone.0329223)
Supplement: Attachment — Submitted filename: Response to Reviewers.pdf [file pone.0329223.s004.pdf]

<u>Response to Reviewers [PONE-D-23-27946R1]</u>

Some simulations of age-period-cohort analysis: Conditions for using random walk model

I thank the reviewers for their constructive comments and suggestions, and apologize for the delay in revising the paper. Below I have quoted the editor and reviewer's comments, followed by my responses.

<u>Additional Editor Comments:</u>

Please address the remaining comments from the reviewers. In particular, the authors are strongly advised to carefully revise the discussions on comparison of model strengths/weaknesses, results and goodness-of-fit and their associated comparisons, and model recommendations. This could potentially address/alleviate the concerns from both reviewers.

[Response]

I thank the additional editor for pointing out the direction for revision. I added Simulation 2 and Simulation 3 to respond to the important comments made by the reviewer 2 and reviewer 4. Specifically, this paper showed the simulations not only where no model can recover the artificial parameters but also where the random walk model performs well. As a result, I think that this paper could clearly describe the discussions on comparison of model strengths/weaknesses and model recommendations.

<u>Reviewer #1:</u>

The author responded to all of my comment, and most of them were addressed. Thank you for the revision. I confirmed it.

[Response]

I thank the reviewer for confirming revisions of this manuscript.

<u>Reviewer #2:</u>

I would change the title to this paper, since I think its emphasis is on the random walk model that uses Bayesian regularization rather than the Random Effects and Ridge Regression models that use Bayesian regularization. The current title is: Systematic simulation of age-period-cohort analysis: Demonstrating bias of Bayesian regularization. But the focus is on comparing three methods that use Bayesian regulation and finding one of them is the preferred model at least for the simulations performed.

[Response]
I appreciate the reviewer's suggestion and changed the title to: Some simulations of age-period-cohort analysis: Conditions for using random walk model.

These statements remind me of my concern in the earlier version of this paper. That the non-linear effects might play a crucial role in the simulations in terms of which models are favored and that this also the case with the linear effects and differential size and larger size. Certainly the author seems to imply that this is more than a study of the problem with the indexes.

We already knew of the effects of the relative number of categories (the size of indexes) on Random Effects models (O'Brien 2017 and Luo and Hodges 2022). That is if there are two random effects and one of them is cohorts they tend to make the linear trend of the cohort effect closer to zero. This is due to the index problem (the cohort factor has more individual categorical variables than the period factor. It has been noted (e.g., O'Brien 2013) that the linear effect of cohort is expected to be close to zero for the IE. So these biases are known.

So it would be important to see other combinations in the simulations. Larger and varied linear effects and larger and varied non-linear effect combinations. You could have combinations in some reasonable range selected randomly and see how the models do with different types of parameters, rather than the uniform and low value linear and non-linear affects you examine in your simulations. Here is a quote from Luo and Hodges 2020: "As m grows—that is, as the true nonlinear component of C grows in magnitude while the true linear component is held constant—the estimation machinery eventually stops attributing all of C's true linear component to A and P." This is interesting given your simulations do not vary the magnitudes of the linear and non-linear effects.

[Response]
I appreciate the important suggestions and have added two simulations to this manuscript. Specifically, Simulation 2 randomly generates the amounts of change in the linear and nonlinear components according to normal distributions, and Simulation 3 sets the artificial parameters so that the pattern with only linear components is less likely to appear (lines 337-339).

In addition, I wrote the results of the above simulations as follows. Simulation 2 showed none of the models can recover the artificial parameters. Therefore, the mitigation of underestimating the linear component of the cohort effects, mentioned in the previous study, does not determine which model performs well without a priori information. In Simulation 3, as the pattern with only linear components was unlikely to appear, the random walk model had less bias than the other models (lines 350-355).

Furthermore, the random walk model performed well for the following reasons: the setting in Simulation 3, where the pattern containing only linear components is unlikely to appear, is consistent with the assumption of Bayesian regularization using the nonlinear components to estimate the linear

components. While the ridge regression model uses λ that is common to the three effects, the nonlinear components of the three effects determine the lower bounds of σ^A, σ^P, and σ^C in the log prior probabilities of the random effects model and the random walk model, and their sigmas affect the assignment of the linear components. Furthermore, Table 2 shows that the random effects model underestimates the linear components of the cohort effects than the random walk model. As a result, the random walk model in Simulation 3 recovered the artificial parameters generated by trigonometric functions rather than random walks (lines 360-370).

Finally, I revised the discussion in this paper as follows: the random walk model implicitly uses the a priori information that the pattern with only linear components, which causes the identification problem, is unlikely to appear (lines 373-375).

Reviewer #3:

This paper is a modest improvement over the original. I am hoping that building on this work we can get comparisons of competing model on actual data.

[Response]

I thank the reviewer for confirming revisions of this manuscript.

Reviewer #4:

I thank the authors for their clarification, and for the addition of some somewhat cautious words regarding all the methods presented. It has improved the paper.

I think I should clarify. I am not saying that I think the random effects or ridge regression models are good at recovering the parameters. Rather I am saying that ALL APC models make assumptions that mean they will produce the wrong results in some circumstances, and those circumstances cannot be known by an applied researcher a priori. And I'm saying that NO APC model should be used without making clear assumptions based on theory.

You have presented some situations in which one particular method performs better than some other methods. You have outlined some mathematical reasons for that. Which is all fine and interesting from a mathematical point of view. But fundamentally, all of these models are just choosing a different point on the line of solutions (Fosse & Winship, 2019b, 2019a; O'Brien, 2021) for the linear component of the APC effects, for mathematical and not substantive reasons. That is, they are all making strong assumptions that are not theoretically informed. It just so happens that those mathematical reasons hit on the true values more often than not with the random walk model. That doesn't mean that the model is "relatively better".

The authors note that "I do not think there is enough scientific evidence for the reviewer's comment that “It’s just that the non-linear components chosen in the simulation happen to chime well with the way that method estimates things."” and suggests I provide mathematical reasons for it. But my reason is logical rather than mathematical: (a) the linear APC components combine to produce identical datasets, (b) the results the model produces are different, and so (c) the differences must be because of the non-linear differences in the DGPs. I don't need a mathematical proof for it to be evident that (a) + (b) -> (c).

[Response]

I appreciate the important comments and have added two simulations to this manuscript. Among them, Simulation 2 reproduced the reviewer's comments that ALL APC models make assumptions that mean they will produce the wrong results in some circumstances. Specifically, Simulation 2 randomly generates the amounts of change in the linear and nonlinear components according to normal distributions (lines 247-248). As a result, unlike Simulation 1, the random walk model did not perform well in Simulation 2. The reason is that different artificial parameters generate the same artificial data, as shown in Fig 1. For example, the artificial parameters shown in Fig 7 generate the artificial data of case 3, meaning that the random walk model in this case incorrectly assigns the linear component to the cohort effects that include the nonlinear components. Therefore, without a priori information, it is impossible to decide which model performs well (lines 306-312).

That begs the question: what is the recommendation of this paper for applied researchers? Should researchers use the random walk method? That seems like a bad recommendation, given it often won't produce the correct results as shown in the simulations, and it is unknowable to the applied researcher (who hasn't simulated the data) whether those situations are occuring in their data. Or are you arguing that none of the methods should be used? If so, that needs to be made more clearly. If it's purely mathematical, that's fine too. But in that case, that needs to be made clear as well, and the recommendation that none of these models should be used without theoretically informed assumptions should be made clearly at all stages of the paper.

Papers like this have a long history of being misinterpreted by researchers, and I'm sure the authors are keen to avoid that happening. Whilst you do note some cautious language, you also state a number of times that the random walk model "may be relatively better". That's true in the simulations you've provided, but I'm sure not in others, because it cannot be for the reasons stated above and in my previous review.

[Response]

I thank the reviewer for the important comments. This paper does not recommend the random walk model under all conditions but recommends using it rather than the other two models when a data generating process with only linear components is unlikely to appear, as shown in Simulation 3.

Specifically, Simulation 3 sets the artificial parameters so that the pattern with only linear components is less likely to appear (lines 338-339). As a result, in Simulation 3, as the pattern with only linear components was unlikely to appear, the random walk model had less bias than the other models (lines 353-355).

Furthermore, the random walk model performed well for the following reasons: the setting in Simulation 3, where the pattern containing only linear components is unlikely to appear, is consistent with the assumption of Bayesian regularization using the nonlinear components to estimate the linear components. While the ridge regression model uses λ that is common to the three effects, the nonlinear components of the three effects determine the lower bounds of σ^A, σ^P, and σ^C in the log prior probabilities of the random effects model and the random walk model, and their sigmas affect the assignment of the linear components. Furthermore, Table 2 shows that the random effects model underestimates the linear components of the cohort effects than the random walk model. As a result, the random walk model in Simulation 3 recovered the artificial parameters generated by trigonometric functions rather than random walks (lines 360-370).

Finally, a problem of the previous manuscript is that it did not describe what a priori information the random walk model uses. Therefore, I revised the discussion in this paper as follows: the random walk model implicitly uses the a priori information that the pattern with only linear components, which causes the identification problem, is unlikely to appear (lines 373-375).
