# Supplementary material for: Some simulations of age-period-cohort analysis applying Bayesian regularization: Conditions for using random walk model
Source: PLoS One. 2025 Aug 8;20(8):e0329223. doi: 10.1371/journal.pone.0329223 (PMC12334005; doi:10.1371/journal.pone.0329223)
Supplement: Attachment — Submitted filename: Response_to_Reviewers_auresp_1.pdf [file pone.0329223.s007.pdf]

<u>Response to Reviewers [PONE-D-24-51931]</u>

"Some simulations of age-period-cohort analysis applying bayesian regularization: Conditions for using random walk model"

I am delighted to receive an invitation to revise and resubmit my manuscript for possible publication in PLOS ONE. I thank the referees for their constructive comments and suggestions. Below I have quoted the reviewer's comments, followed by my responses.

<u>Reviewer #1</u>

1. General

The manuscript investigates the identification problem in age-period-cohort (APC) analysis, a common issue in time-series analysis across various fields. The author focuses on three Bayesian models used to address this problem: the random effects model, the ridge regression model, and the random walk model. Across three simulations using artificial data, the paper aims to determine the conditions under which the random walk model is preferable, specifically in context to its ability to mitigate the underestimation of the linear component of cohort effects.

The manuscript has several key strengths, including its focus on the highly relevant and well-recognized identification problem inherent in APC analysis. The methodology is clearly articulated, with the simulation design and the specifications of the models described in sufficient detail. The author's approach of using simulations to compare the performance of different models is appropriate and valuable. Finally, the manuscript benefits from a well-structured and logical organization, enhancing its overall readability and accessibility.

[Response]

I thank the reviewer for the positive comments.

However, the manuscript's ability to generalize the results may be limited due to the specific parameters used in simulations. The analysis and the specific recommendations for model selection could be strengthened. The author needs to clarify the limitations of the study, refine the conclusions, and provide a more nuanced discussion of the role of a priori information. I recommend that the manuscript be considered for publication after addressing these significant revisions.

[Response]

I appreciate these important suggestions. I revised the conclusion of this paper to be more cautious.

Line 377-380: “the simulations using trigonometric functions for the nonlinear components showed that the random walk model recovered artificial parameters better than the other models, if the pattern with only linear components is unlikely to appear.”

Then, this paper removed “the pattern containing only linear components is less likely to appear than other patterns in real data” and the discussion of a priori information. Moreover, I added a discussion of polynomial functions as data generating processes other than trigonometric functions in “Applicability” subsection.

2. Abstract

The abstract adequately summarizes the central research question, the methodological approach, and the primary findings of the study. However, its impact could be strengthened by including a more specific description of the conditions under which the random walk model demonstrates “superior” performance.

Further, this section in particular could benefit from several improvements in clarity and precision. Certain phrases, such as the description of how multilevel analysis impacts cohort effects, are awkwardly phrased and could be reworded for smoother flow. Vague language, particularly regarding the “various patterns” of simulations used in previous studies, should be clarified with more specific details. Furthermore, breaking down some of the longer sentences would improve overall readability and ensure the abstract’s key messages are easily grasped by the reader.

For example, “Applying multilevel analysis results in the linear component of the cohort effects close to zero and the intrinsic estimator is sensitive to the coding of the design matrix” is a bit awkward and could be rephrased for better flow.

[Response]

As per the comments, I made some revisions to improve readability, such as deleting vague language and breaking down some of the longer sentences.

3. Introduction

The introduction effectively establishes the context and rationale for the study. It clearly defines the APC identification problem, highlighting its significance in time-series analysis. The limitations of current approaches are succinctly explained, adequately justifying the need for the comparative analysis presented.

[Response]

I thank the reviewer for the positive comments.

4. Theory

The Theory section is a strong point of the manuscript, providing a solid foundation for understanding the complexities of APC analysis and the proposed solutions. The section then clearly delineates the three Bayesian models under consideration outlining their mathematical formulations and underlying assumptions. Particularly valuable is the detailed discussion of linear and non-linear components and the impact of index weights. This section provides useful insights into the mechanisms driving the differences in model performance, particularly highlighting why the random walk model might mitigate the underestimation of the linear component of cohort effects under specified simulated conditions.

[Response]

I thank the reviewer for the positive comments.

Line 170-172: "However, Sakaguchi and Nakamura [11] suggested that the random walk model performs well compared to the random effects model; consequently, this paper examines the impact of the index weights." The phrase "performs well" is vague.

[Response]

I revised the above ambiguous sentence to the following.

Line 170-175: "Here, Sakaguchi and Nakamura [11] suggested that the index weights of the random effects model had a greater impact than those of the random walk model. However, the previous study did not verify that the index weights of the random effects model were large regardless of the values of I or J. Consequently, this paper compares the impact of the index weights in Eq (13) and Eq (14)."

5. Methods

The Methods section provides a clear description of the simulation design. The rationale behind the chosen simulation parameters and the overall approach is well-justified. The decision to use trigonometric functions for generating non-linear components is clearly explained, and while acknowledging potential limitations in generalizability, the author provides a reasonable justification for this choice within the context of the study. Furthermore, the bias evaluation function, based on the parameter 's', is appropriate for assessing the models' ability to recover the true underlying parameters and provides a quantifiable metric for comparison. However, explicitly acknowledging that different data-generating mechanisms might yield different results, and briefly explaining why trigonometric functions were chosen over other potential functions, could enhance this section.

[Response]

I appreciate these important suggestions. I added a discussion of polynomial functions and why trigonometric functions are preferable to polynomial functions.

Line 190-195: "There are some candidates for data generating processes, including polynomial functions. However, analysts cannot freely determine each component of artificial parameters generated by polynomial functions, because they contain not only linear components but also nonlinear components. Thus, this paper uses trigonometric functions for the nonlinear components, as they contain no linear components and can be set to any amount of change."

6. Results

The Results section effectively communicates the findings of each simulation, employing a clear and organized presentation style. The interpretation of the results is generally sound and logically follows from the data presented. However, the discussion of the results could be improved by more strongly emphasizing the limitations of the study, particularly the potential for limited generalizability due to the specific parameter settings used in the simulations. While the results offer valuable insights, a more explicit acknowledgement of these limitations would provide a more balanced and nuanced interpretation of the findings - especially for applied researchers.

[Response]

I appreciate these important suggestions. I removed the following sentence from "Simulation 3" subsection because the previous manuscript suggested an overgeneralization in real-world data: "this is because mixing complex factors that affect real data does not result in only linear components, such as age effects continuing to increase linearly." Therefore, I revised the purpose of Simulation 3 to consider the assumptions of Bayesian regularization. I also revised the conclusions of this paper to be more cautious, not generalizing, and focusing only on trigonometric functions and polynomial functions.

7. Discussion

The Discussion section summarizes the main findings of the study and begins to explore their implications for APC analysis. It connects the results back to the research questions and provides an initial interpretation of their significance. However, the discussion needs to be significantly expanded in several key areas. Specifically, the limitations of the study, particularly the potential for limited generalizability due to the specific parameters used in the simulation, deserve a more thorough treatment. For example, are there specific types of data or research questions where

these limitations are particularly problematic? Further, while acknowledging that the random walk model "performed well" under the simulated conditions, the discussion should provide a more nuanced perspective on the applicability of these findings to real-world data (and possibly suggest avenues for future research to further investigate these limitations and enhance external validity). Also, the role of a priori information and its influence on model selection should be discussed in greater depth, for example, addressing how it might affect the performance of each model.

[Response]

I appreciate these important suggestions. I revised the conclusion of this paper to be more cautious.

Line 377-380: "the simulations using trigonometric functions for the nonlinear components showed that the random walk model recovered artificial parameters better than the other models, if the pattern with only linear components is unlikely to appear."

Then, this paper removed "the pattern containing only linear components is less likely to appear than other patterns in real data" and the discussion of a priori information, to avoid generalizations in real-world data.

Moreover, I added the discussion of polynomial functions as data generating processes other than trigonometric functions in "Applicability" subsection. Then, this paper shows how to generate artificial parameters for polynomial functions so that future research can investigate them. Finally, I added the following to the conclusion of this paper.

Line 398-403: "As in Simulation 3, the absence of dots in the gray area indicates that the polynomial function does not generate the artificial parameter containing only linear components. Fig 9 shows that there are many dots in the gray area at $H = 2$ and no dots at $H = 8$. The reason is that w_1 affects only the linear component, while w_3 and w_5 affect not only the linear component but also the nonlinear components."

Line 405-410: "In summary, the polynomial functions in this subsection are less likely to generate the pattern with only linear components as the degree of the polynomial increases. Therefore, the random walk model may have a smaller bias than the other models even when data generating processes can be approximated by polynomial functions with a large degree of polynomial."

Line 378-380: "For the above reason, this paper expects the random walk model to perform relatively better than the other two models in real data if there is no other strong a priori information [20]." This conclusion is too broad. The simulations demonstrate the random walk model's advantage under specific conditions, but more evidence is needed to support its general superiority in the absence of strong a priori information. The author should explicitly state that the conclusion applies to scenarios similar to Simulation 3, where only linear components are

unlikely. A more cautious conclusion would focus on the specific circumstances where the random walk model demonstrated an advantage in the simulations. It is more accurate to conclude that the random walk model is advantageous when there are both linear and non-linear components for each age, period, and cohort.

[Response]

As per the comments, I revised the conclusions of this paper to be more cautious, not generalizing, and focusing only on trigonometric functions and polynomial functions.

Line 381-383: "Finally, this paper has several limitations, such as only discussing the main three models applying Bayesian regularization, not changing the indexes, for example I = 12 or J = 8, and not considering nonlinear components other than trigonometric functions." This accurately describes the limitations of the paper. The author should therefore expand the discussion of these limitations in the discussion section.

[Response]

I thank the reviewer for the comments. This paper modified to discuss trigonometric and polynomial functions rather than limitations on generalization to real-world data.

8. Tables & Figures

The figures and tables are, for the most part, well-constructed and contribute to the clarity and interpretability of the results. The tables provide a concise and well-organized summary of the simulation results, facilitating a quick comparison across models and conditions. However, the effectiveness of some figures could be enhanced by providing more descriptive captions. Currently, the captions are brief and could benefit from additional details to make them self-explanatory.

For example, instead of "Artificial data with only linear components (Simulation 1)", it could be "Simulation 1: Artificial data generated with only linear components for age, period, and cohort effects. Each panel represents a different combination of linear slopes as described in Table 2."

[Response]

As per the comments, this paper revised the captions of Fig 1 and Fig 2.

__Reviewer #2__

1) The paper is well-structured, providing a clear breakdown of the theory, methods, and results. However, the writing can be improved for better readability. Some sentences, particularly in the introduction and discussion, are long and complex, making comprehension difficult. Simplifying them would enhance clarity.

[Response]

As per the comments, I revised the "Introduction" and "Discussion" section to improve readability, such as breaking down some of the longer sentences.

2) Consider adding a brief overview at the end of the introduction to explicitly state the key findings. This will help guide the reader through the study.

[Response]

I thank the reviewer for the comments. This paper included a brief overview in "Abstract" section because of the lengthy "Introduction" section as described above.

3) The study compares three Bayesian regularization models (random effects, ridge regression, and random walk) in the context of APC analysis. While the motivation for the study is clear, the justification for the choice of trigonometric functions for nonlinear components could be elaborated. Why were these functions selected over other nonlinear patterns?

[Response]

I appreciate the important comments. I added a discussion of polynomial functions and why trigonometric functions are preferable to polynomial functions.

Line 190-195: "There are some candidates for data generating processes, including polynomial functions. However, analysts cannot freely determine each component of artificial parameters generated by polynomial functions, because they contain not only linear components but also nonlinear components. Thus, this paper uses trigonometric functions for the nonlinear components, as they contain no linear components and can be set to any amount of change."

4) The definition of bias evaluation function is useful, but it would be beneficial to discuss any potential limitations or assumptions associated with its use.

[Response]

I thank the reviewer for the comments. The limitation of the bias evaluation functions is that we need to know data generation processes. In other words, we can use this function for simulations, but not

for real-world data.

5) The study presents three simulations to analyze model performance.
- Simulation 1 effectively illustrates the impact of index weights on underestimation of cohort effects.
- Simulation 2 highlights the challenges in parameter recovery but does not fully explore why none of the models performed well. Could additional constraints or adjustments improve the performance?
[Response]
I thank the reviewer for the comments. The reasons why none of the models performed well in Simulation 2 are as follows.
Line 311-312: "The reason is that different artificial parameters generate the same artificial data, as shown in Fig 1."
Therefore, it is difficult to perform well by adding any constraints to the models, but imposing constraints on the artificial parameters, as in Simulation 3, may improve the performance.

- Simulation 3 shows that the random walk model performs better when only linear components are unlikely to appear. However, the assumption that such patterns are less likely in real data should be supported with references or empirical examples.
[Response]
I appreciate the important comments. I removed the following sentence from "Simulation 3" subsection because the previous manuscript suggested an overgeneralization in real-world data: "this is because mixing complex factors that affect real data does not result in only linear components, such as age effects continuing to increase linearly." Moreover, I added a discussion of polynomial functions as data generating processes other than trigonometric functions in "Applicability" subsection. The reason for adding the discussion of polynomial functions to this paper is as follows.
Line 398-403: "As in Simulation 3, the absence of dots in the gray area indicates that the polynomial function does not generate the artificial parameter containing only linear components. Fig 9 shows that there are many dots in the gray area at $H = 2$ and no dots at $H = 8$. The reason is that w_1 affects only the linear component, while w_3 and w_5 affect not only the linear component but also the nonlinear components."
Line 405-410: "In summary, the polynomial functions in this subsection are less likely to generate the pattern with only linear components as the degree of the polynomial increases. Therefore, the random walk model may have a smaller bias than the other models even when data generating processes can

be approximated by polynomial functions with a large degree of polynomial.”

6) The conclusion suggests that the random walk model performs better under certain conditions. It would be helpful to specify the types of datasets or research questions where this model would be most applicable.

[Response]

I appreciate the important suggestions. I added the discussion of polynomial functions as data generating processes where the random walk model may be applicable. The reason is that the polynomial functions in this paper are less likely to generate the pattern with only linear components as the degree of the polynomial increases.
