# Supplementary material for: Some simulations of age-period-cohort analysis applying Bayesian regularization: Conditions for using random walk model
Source: PLoS One. 2025 Aug 8;20(8):e0329223. doi: 10.1371/journal.pone.0329223 (PMC12334005; doi:10.1371/journal.pone.0329223)
Supplement: Attachment — Submitted filename: Response_to_Reviewers_auresp_2.pdf [file pone.0329223.s008.pdf]

<u>Response to Reviewers [PONE-D-24-51931R1]</u>

"Some simulations of age-period-cohort analysis applying bayesian regularization: Conditions for using random walk model"

<u>Reviewer #1</u>

The author has attended to all the recommendations appropriately. The author has made an effort to address the "cautious conclusion" and "generalizability" points by modifying the wording in the conclusion and adding a discussion on polynomial function. Furthermore, instances of ambiguity and vague language have been clarified. The addition of the section on "Applicability" has strengthened the paper.

[Response]

I thank the reviewer for the positive comments.

<u>Reviewer #3</u>

The author has addressed all review comments from the first round. I suggest one minor revision: including visualizations of a toy dataset to help readers better understand the type of data being considered in this paper.

[Response]

I thank the reviewer for the important suggestions. As per the comments, I added Table 3, which visualizes the data setting considered in the simulations of this paper. Moreover, I added the following to this paper.

Line 345-346: Table 3 briefly summarizes the settings for the amount of change in each component of Simulation 1 to 3.

Table 3. Settings for the amount of change in each component (Simulation 1 to 3)

	Linear component	Nonlinear component
Simulation 1	Three types: 0, 0.1, -0.1	One-half of the linear component
Simulation 2	Normal (0, 0.1)	Normal (0, 0.1)
Simulation 3	Normal (0, |the nonlinear component|)	Normal (0, 0.1)
