# Supplementary material for: Some simulations of age-period-cohort analysis applying Bayesian regularization: Conditions for using random walk model
Source: PLoS One. 2025 Aug 8;20(8):e0329223. doi: 10.1371/journal.pone.0329223 (PMC12334005; doi:10.1371/journal.pone.0329223)
Supplement: Attachment — Submitted filename: Comments to the Editorial Office.docx [file pone.0329223.s005.docx]

Major Revision of Revised Manuscript

Dear Associate Editor

I hope this email finds you well.

I have reviewed the revised manuscript **PONE-D-24-51931** titled “**Some simulations of age-period-cohort analysis: Conditions for using random walk model** " and somewhat assessed the authors' responses to the comments. The manuscript requires substantial improvements in justifications, clarity, and result interpretations, but it does not need fundamental methodological changes. If the authors address these issues thoroughly, the paper could be a strong contribution to APC modeling research. I recommend a **major revision** based on my attached recommendations.

Thank you for the opportunity to contribute to the review process. Please let me know if any further input is required.

Best regards,
B.Bidar
Reviewer, PLOS ONE Journal
Department of Chemical Engineering,
Shahid Nikbakht Faculty of Engineering,

University of Sistan and Baluchestan

Zahedan, Iran
[b.bidar@eng.usb.ac.ir](mailto:b.bidar@eng.usb.ac.ir)
