# Supplementary material for: Some simulations of age-period-cohort analysis applying Bayesian regularization: Conditions for using random walk model
Source: PLoS One. 2025 Aug 8;20(8):e0329223. doi: 10.1371/journal.pone.0329223 (PMC12334005; doi:10.1371/journal.pone.0329223)
Supplement: Attachment — Submitted filename: Recommendations.docx [file pone.0329223.s006.docx]

Review of the paper with manuscript ID: PONE-D-24-51931

"**Some simulations of age-period-cohort analysis: Conditions for using random walk**

**model**"

The manuscript addresses an important issue in age-period-cohort analysis, specifically the identification problem and how different Bayesian regularization methods handle it. The concerns and suggestions of reviewers have been partially implemented. The title of paper in now acceptable and more it is more relevant to the topic of the paper than the previous title. I think some parts still need to be revised to become a standard paper.

Comments to authors:

1) The paper is well-structured, providing a clear breakdown of the theory, methods, and results. However, the writing can be improved for better readability. Some sentences, particularly in the introduction and discussion, are long and complex, making comprehension difficult. Simplifying them would enhance clarity.
2) Consider adding a brief overview at the end of the introduction to explicitly state the key findings. This will help guide the reader through the study.
3) The study compares three Bayesian regularization models (random effects, ridge regression, and random walk) in the context of APC analysis. While the motivation for the study is clear, the justification for the choice of trigonometric functions for nonlinear components could be elaborated. Why were these functions selected over other nonlinear patterns?
4) The definition of bias evaluation function is useful, but it would be beneficial to discuss any potential limitations or assumptions associated with its use.
5) The study presents three simulations to analyze model performance.

- Simulation 1 effectively illustrates the impact of index weights on underestimation of cohort effects.
- Simulation 2 highlights the challenges in parameter recovery but does not fully explore why none of the models performed well. Could additional constraints or adjustments improve the performance?
- Simulation 3 shows that the random walk model performs better when only linear components are unlikely to appear. However, the assumption that such patterns are less likely in real data should be supported with references or empirical examples.

6) The conclusion suggests that the random walk model performs better under certain conditions. It would be helpful to specify the types of datasets or research questions where this model would be most applicable.
